# Risk Factors of Sudden Cardiac Arrest during the Postoperative Period in Patient Undergoing Heart Valve Surgery

**DOI:** 10.3390/jcm11237098

**Published:** 2022-11-30

**Authors:** Piotr Duchnowski

**Affiliations:** Cardinal Wyszynski National Institute of Cardiology, Alpejska 42, 04-628 Warsaw, Poland; pduchnowski@ikard.pl

**Keywords:** sudden cardiac arrest, N-terminal of the prohormone brain natriuretic peptide predicts postoperative (NT-proBNP), valve surgery

## Abstract

Background: Sudden cardiac arrest (SCA) is the sudden cessation of normal cardiac activity with hemodynamic collapse. This usually leads to sudden cardiac death (SCD) when cardiopulmonary resuscitation is not undertaken. In patients undergoing heart valve surgery, postoperative SCA is a complication with a high risk of death, cerebral hypoxia and multiple organ dysfunction syndrome (MODS). Therefore, knowledge of the predictors of postoperative SCA is extremely important as it enables the identification of patients at risk of this complication and the application of the special surveillance and therapeutic management in this group of patients. The aim of the study was to evaluate the usefulness of selected biomarkers in predicting postoperative SCA in patients undergoing heart valve surgery. Methods: This prospective study was conducted on a group of 616 consecutive patients with significant valvular heart disease that underwent elective valve surgery with or without coronary artery bypass surgery. The primary end-point at the intra-hospital follow-up was postoperative SCA. The secondary end-point was death from all causes in patients with postoperative SCA. Patients were observed until discharge from the hospital or until death. Logistic regression was used to assess the relationships between variables. Results: The postoperative SCA occurred in 14 patients. At multivariate analysis, only NT-proBNP (odds ratio (OR) 1.022, 95% confidence interval (CI) 1.012–1.044; *p* = 0.03) remained independent predictors of the primary end-point. Age and NT-proBNP were associated with an increased risk of death in patients with postoperative SCA. Conclusions: The results of the presented study indicate that SCA in the early postoperative period in patients undergoing heart valve surgery is an unpredictable event with high mortality. The potential predictive ability of the preoperative NT-proBNP level for the occurrence of postoperative SCA and death in patients after SCA demonstrated in the study may indicate that the overloaded and damaged myocardium in patients undergoing heart valve surgery is particularly sensitive to non-physiological conditions prevailing in the perioperative period, which may cause serious hemodynamic disturbances in the postoperative period and lead to death.

## 1. Introduction

Sudden cardiac arrest (SCA) is by definition the cessation of mechanical heart function, which occurs within 1 h of the onset of new or worsening symptoms. It usually leads to sudden cardiac death (SCD) when cardiopulmonary resuscitation is not undertaken. The main recognized reasons for the occurrence of SCA are: a canalopathies and cardiomyopathies as well as myocarditis and coronary anomalies that predominate among the young. Over the age of 40, SCD is largely associated with the presence of structural diseases of the heart muscle, among which coronary heart disease predominates, in addition to valvular heart disease and heart failure [1,2,3,4,5]. The prediction of SCA is the philosopher’s stone of arithmology. It is now known that the propensity to develop sudden cardiac arrhythmias that may lead to SCA (such as asystole, pulseless electrical activity, ventricular fibrillation or ventricular tachycardia) is associated with an unfavorable coincidence of interaction of a susceptibility substrate (e.g., genetic or acquired changes in the electrical or mechanical properties of the heart) with many temporary factors [1,2,6]. To date, the only indicator that shows a consistent relationship with an increased risk of SCA and left ventricular (LV) dysfunction is left ventricular ejection fraction (LVEF). Other new parameters of promise for SCD prediction include biochemical indices such as high-sensitivity Troponin T (hs-TnT) and the concentration of BNP, for which encouraging results were obtained in preliminary studies. The postoperative SCA in patients undergoing heart valve surgery is a complication which significantly increases the risk of hospital death as well as cerebral hypoxia and multiple organ dysfunction syndrome (MODS) [7,8,9,10,11]. Knowledge of the predictive factors of postoperative SCA is very important because it enables the preoperative selection of patients at risk of this complication, particular attention during the process of qualifying for interventional treatment, vigilant supervision of the patient in the perioperative period and taking an immediate response in the event of any warning symptoms. Therefore, we made an attempt to check the usefulness of selected biomarkers in predicting the occurrence of SCA in the early postoperative period in patients with heart valve disease undergoing cardiac surgery.

## 2. Methods

The current prospective study was performed on consecutive patients with hemodynamically significant valve defects who underwent elective replacement or repair of the valve with or without additional procedures at the Cardinal Wyszynski National Institute of Cardiology in Warsaw, Poland. Patients under 18 years of age, unwilling to participate, or diagnosed with active malignancy, autoimmune diseases, chronic inflammatory bowel disease and significant atherosclerotic lesions in the carotid arteries were excluded from the study. The day before surgery a blood sample for biomarkers was collected from each patient. Full blood counts were measured from K2EDTA samples using a Sysmex K-4500 (Sysmex, Kobe, Japan) electronic counter. The plasma levels of NT-proBNP concentrations were measured by electrochemiluminescent immunoassays Elecsys 2010 (Roche, Munich, Germany). All treatments were performed through median sternotomy under general anesthesia and normothermic conditions. Cold cardioplegia was used in each case. The primary endpoint in in-hospital follow-up is SCA during the early postoperative period, defined as cardiac arrest within one hour of the onset of acute symptoms, regardless of whether it was reversed by resuscitation. The secondary end-point was death from all causes in patients with postoperative SCA. The observation period was until discharge from hospital or death. The Institutional Ethics Committee approved the study protocol, number 1705.

## 3. Statistical Analysis

All analyses were performed using IBM SPSS software. The collected data are presented as mean ± SD and frequency (%). Comparisons between individual groups of variables were performed using the Mann–Whitney U test, the Pearson χ^2^ test or the Student’s *t* test. The Shapiro–Wilk normality test was used to test the sample distribution. Logistic regression was used to assess relationships between variables. The following covariates were investigated for association with the primary and secondary end-points in univariate analysis: age, aortic cross-clamp time, cardiopulmonary bypass time, atrial fibrillation, body mass index, chronic obstructive airway disease, coronary artery disease, coronary artery bypass grafting (CABG) procedure, GFR, high-sensitivity troponin T (hs-TnT), hemoglobin, left ventricular ejection fraction (LVEF), the functional class of heart failure according the New York Heart Association (NYHA classes), N-terminal of the prohormone brain natriuretic peptide (NT-proBNP), peripheral atherosclerosis, pulmonary blood pressure, tricuspid annulus plane systolic excursion (TAPSE), aortic valve replacement (AVR), mitral valve plasty (MVP), mitral valve replacement (MVR), AVR plus MVR, and postoperative major blending. Significant determinants (*p* < 0.05) identified on the basis of univariate analysis were subsequently introduced into multivariate models. The Spearman rank correlation analysis was used to search for associations between the preoperative value of NT-proBNP level and LVEF, NYHA classes and hs-TnT.

## 4. Results

This study included 616 patients undergoing heart valve surgery with or without coronary artery bypass surgery. The mean (SD) age in the study population was 63 [12] years. Significantly impaired left ventricular systolic function (ejection fraction ≤ 35%) in the preoperative period was reported in forty-eight (8%) patients. The mean preoperative NT-proBNP level was 1993 pg/mL (standard deviation (SD) ± 1498). Table 1 shows the characteristics of the patients studied. Postoperative SCA occurred in 14 patients (during the monitoring, the ecg was found: ventricular fibrillation in six patients, ventricular tachycardia in four patients, pulseless electrical activity in one patient, and asystole in three patients). The average period from the end of surgery to the occurrence of SCA was 2 days ((SD) ± 1.5). In each case of SCA, cardiopulmonary resuscitation was used, which resulted in the restoration of a hemodynamically stable heart rhythm in 10 patients (among which two patients required permanent pacemaker implantation). In four cases, death occurred despite resuscitation. In a further follow-up with 10 patients who survived SCA, five patients died due to progressive MODS. The statistically significant predictors of postoperative sudden cardiac arrest at univariate and multivariate analyses are presented in Table 2. At multivariate analysis, only NT-proBNP (odds ratio (OR) 1.022; 95% confidence interval (CI) 1.012–1.044; *p* = 0.03) remained an independent predictor of the primary end-point. In the group of patients with SCA, the mean LVEF value was 52% (±14%) and was significantly lower compared to patients with no SCA 58% (±12%) (*p* < 0.05). Moreover, the mean preoperative value of NT-proBNP in patients with SCA was 5346 pg/mL (±3582) and was significantly higher compared to patients with no SCA 1910 pg/mL (±1398) (*p* < 0.05). A significant correlation was found between the level of preoperative NT-proBNP and pre-operative LVEF (r = −0.39; *p* < 0.001), level of hs-TnT (r = 0.38; *p* < 0.001) and NYHA classes (r = 0.41, *p* < 0.001). A secondary end-point occurred in nine patients (1.5% of total patients). Statistically significant predictors of death from all causes in patients with postoperative SCA at univariate analysis were NT-proBNP (OR 1.021; 95% CI: 1.011–1.022; *p* = 0.001) and age (OR 1.086; 95% CI 1.002–1.176; *p* = 0.04) (multivariate analysis was not performed due to an insufficient number of events) (Table 3). The mean NT-proBNP value in the group with postoperative SCA who died was 6978 pg/mL (±3582) and was significantly higher compared to patients with SCA who survived 2410 pg/mL (±943) (*p* < 0.05). The total in-hospital mortality was 3.7%.

Ninety-eight patients (16% of the total group with severe valvular disease) had concomitant coronary artery disease. Of these, 95 patients (15% of the entire group) underwent an additional coronary artery bypass procedure. Importantly, however, no significantly higher NT-proBNP concentration was found in patients with concomitant coronary artery disease (*p* = 0.30) and in patients who underwent additional coronary artery bypass grafting (*p* = 0.33) compared to the remaining group of 518 patients with severe valvular heart without coexisting coronary artery disease. Among the 98 patients with severe valvular heart disease and concomitant coronary artery disease undergoing cardiac surgery, 3 patients experienced sudden cardiac arrest in the early postoperative period. In the univariate logistic regression analysis, both the coexistence of coronary artery disease (*p* = 0.29) and the additional coronary artery bypass procedure (*p* = 0.12) in patients undergoing heart valve surgery were not predictors of sudden cardiac arrest in the early postoperative period.

## 5. Discussion

The occurrence of SCA in the early postoperative period in patients undergoing heart valve surgery is a critical event. The alertness of the medical staff and the reaction time can be decisive in reversing the further fate of the patient. Additionally, the presence of a postoperative wound poses a major challenge for the team undertaking resuscitation in saving the patient’s life. Therefore, the knowledge of predictive factors for SCA in the early postoperative period in this group of patients gives the patient a chance to survive through the special surveillance of patients in the critical postoperative period and early response in the event of predictive symptoms [12,13].

Arrhythmias are a fairly common complication occurring in the early postoperative period in patients undergoing cardiac surgery. The most common arrhythmia in this group of patients is postoperative atrial fibrillation. In turn, sustained ventricular tachycardia and ventricular fibrillation (main direct mechanism of occurrence of postoperative SCA) are quite rare and, ranging from 0.7% to 3.1% according to various investigators, and are associated with a high risk of death [3,14,15,16,17,18,19]. In the present study, SCA occurred in 14 patients, which is approximately 2% of patients undergoing heart valve surgery. Of the 14 patients who experienced SCA, in-hospital death was observed in nine patients.

The present study showed that the preoperative NT-proBNP level may be a potential predictor of SCA and death in the early postoperative period in patients undergoing heart valve surgery. Due to the fact that the active form of BNP is involved in maintaining the homeostasis of the cardiovascular system, NT-proBNP is currently widely used in the diagnosis, assessment of progression, and the degree of myocardial damage. NT-proBNP is mainly released by left ventricular cardiomyocytes in response to myocardial tone and increased intravascular volume. In severe valvular heart disease, there is a significant pressure and/or volume overload of the left ventricular muscle, which leads to an increase in NT-proBNP release from cardiomyocytes. However, long-lasting overload of the heart muscle, may be the cause of a progressive degenerative process associated with slow cardiomyocyte necrosis and the occurrence of fibrosis [20,21,22,23,24,25,26,27,28,29,30,31]. Very high NT-proBNP values present in the blood serum of patients with severe valvular heart disease may indicate significant damage to the overloaded left ventricular muscle, which may be confirmed by the significant correlation shown in the study between the preoperative NT-proBNP concentration and the NYHA class, preoperative hs-TnT level and left ventricular systolic function assessed using LVEF. The potential predictive ability of NT-proBNP levels for the primary and secondary endpoints demonstrated in the study may indicate that the overloaded and damaged myocardium in a patient undergoing heart valve surgery is particularly sensitive to non-physiological conditions during cardiac surgery on cardiac arrest. It seems that the conditions prevailing during extracorporeal circulation may favor further myocardial damage and contribute to the occurrence of electrical disturbances underlying mechanical cardiac arrest. The above thesis may be confirmed by the results of previously published studies, which showed a significant correlation between aortic cross-clamp time and cardiopulmonary bypass time with the postoperative high-sensitivity Troponin T level [3,32,33,34]. In addition, the presence of electrolyte disturbances, such as hypokalemia and hypomagnesaemia, metabolic acidosis, decrease in hemoglobin level, or increased supply of sympathomimetics in the early postoperative period may be a direct factor causing dangerous ventricular arrhythmia in the myocardium damaged by long-term valvular heart disease [15].

It is worth mentioning that out of 616 patients who underwent surgical treatment of valvular heart disease, 98 patients had significant atherosclerotic changes in the coronary arteries and 95 underwent coronary artery bypass grafting at the same time. However, the statistical analysis performed showed that the coexistence of significant stenosis in the coronary arteries does not increase the risk of sudden cardiac arrest in the early postoperative period.

Additionally, although the results of the presented study indicate that the preoperative level of NT-proBNP may be a potential predictor of SCA and death in patients with previous SCA in the early postoperative period, the essence of the above study is the fact that SCA is an unpredictable event with a high risk of death. Both the previously presented studies and the above results indicate a significant problem of preoperative myocardial damage resulting from the long-term impact of severe valvular defects/defects on the heart muscle and thus the possibility of serious events in the postoperative period, including hemodynamic instability, shock, and the possibility of SCA and death in the early postoperative period [3,22,35]. Therefore, the group of patients with severe heart valve disease and very high NT-proBNP values requires special attention and professionalism during surgery and in the postoperative period, including intensive medical supervision and ECG monitoring. In addition, the study results may also suggest that earlier qualification for surgery with less advanced myocardial damage and lower NT-proBNP, or qualification for less burdensome procedures such as TAVI or percutaneous valve repair, may be associated with a reduction in the incidence of severe postoperative complications and death.

## 6. Conclusions

The results of the presented study indicate that SCA in the early postoperative period in patients undergoing heart valve surgery is an unpredictable event with high mortality. The potential predictive ability of the preoperative NT-proBNP level for the occurrence of postoperative SCA and death in patients after SCA demonstrated in the study may indicate that the overloaded and damaged myocardium in a patient undergoing heart valve surgery is particularly sensitive to the non-physiological conditions prevailing in the perioperative period, which may cause serious hemodynamic disturbances in the postoperative period, and consequently, may be the cause of death. In future studies, enlarging the group may enable confirming the obtained results. In addition, the extension of the study will also enable confirming whether the coexistence of ischemic heart disease is conducive to sudden cardiac arrest in the early postoperative period. Knowledge of the predictors of postoperative complications is extremely important because it allows the implementation of an appropriate perioperative strategy, which in turn can improve treatment outcomes in patients with valvular heart disease.

## Figures and Tables

**Table 1:** Baseline characteristics of the study population.

Preoperative Characteristics of Patients (*n* = 616)	Values
Age, years *	63 ± 12
Atrial fibrillation, *n* (%)	268 (43%)
Coronary artery disease, *n* (%)	98 (15%)
Chronic kidney disease (GFR < 60 mL/min/1.73 m^2^), *n* (%)	196 (31%)
Creatinine, mg/dL *	0.8 ± 0.5
Hemoglobin, g/dL *	13.7 ± 1.4
LV ejection fraction, (%) *	57 ± 12
Male: men, *n* (%)	355 (58%)
NYHA, (classes) *	2.6 ± 0.6
Hs-TnT, ng/L *	26 ± 21
Nt-proBNP, pg/mL *	1993 ± 1498
Intraoperative and postoperative characteristics of patients	Values
Aortic cross-clamp time, min *	101 ± 41
Cardiopulmonary bypass time, min *	132 ± 53
The day of sudden cardiac arrest, days *	2 ± 1.5
Postoperative major blending, *n* (%)	45 (7%)
Main procedures:	
AVR, *n* (%)	319 (52%)
AVR + MVR, *n* (%)	54 (9%)
AVP, *n* (%)	17 (3%)
MVR, *n* (%)	111 (18%)
MVP, *n* (%)	115 (18%)
Concomitant procedures:	
CABG, *n* (%)	95 (15%)
TVP, *n* (%)	115 (18%)

Values are represented by the mean * and a measure of the variation of the internal standard deviation. Abbreviations: AVP = aortic valve plasty; AVR = aortic valve replacement; CABG = coronary artery bypass grafting; MVP = mitral valve plasty; MVR = mitral valve replacement; GFR = glomerular filtration rate; LV = left ventricle; NYHA = New York Heart Association; TVP = tricuspid valve plasty.

**Table 2:** Analysis of predictive factors for the occurrence of postoperative sudden cardiac arrest.

		Univariable			Multivariable	
Variable	Odds Ratio	95% Cl	*p*-Value	Odds Ratio	95% Cl	*p*-Value
Nt-proBNP, pg/mL	1.026	1.009–1.046	0.003	1.022	1.012–1.044	0.03
NYHA, classes	3.255	1.254–8.454	0.01			

Abbreviations: Nt-proBNP = N-terminal of the prohormone brain natriuretic peptide, NYHA = New York Heart Association. Univariate analysis followed by multivariate regression analysis was performed.

**Table 3:** Univariate analysis of predictive factors for the occurrence of death in patients with SCA.

Variable	Odds Ratio	95% Cl	*p*-Value
Nt-proBNP, pg/mL	1.021	1.011–1.022	0.003
Age, years	1.086	1.002–1.176	0.04

Abbreviation: Nt-proBNP = N-terminal of the prohormone brain natriuretic peptide. Multivariate analysis was not performed due to insufficient number of events.

## Data Availability

Research data available from the author of the publication.
